# A pair of new sister species of *Loneura* (Psocodea, ‘Psocoptera’, Ptiloneuridae) from Valle del Cauca, Colombia, representing a new infrageneric group

**DOI:** 10.3897/zookeys.168.2508

**Published:** 2012-01-31

**Authors:** Alfonso N. García Aldrete, Julián A. Mendivil Nieto, Ranulfo González Obando

**Affiliations:** 1 Departamento de Zoología, Instituto de Biología, Universidad Nacional Autónoma de México, Apartado Postal 70-153, 04510 México, D. F., MÉXICO; 2 Departamento de Biología, Facultad de Ciencias Naturales y Exactas, Universidad del Valle, Santiago de Cali, COLOMBIA

**Keywords:** South America, classification of *Loneura*, species richness

## Abstract

Two sister species of *Loneura*, from Valle del Cauca, Colombia, are here described and illustrated. They constitute a new species group that modifies the scheme of classification, proposed earlier for the genus by García Aldrete et al. (2011b). The new group is characterized by having the central sclerite of the male hypandrium with four posterior projections. A key to the males of Group II is included. The types are deposited in the Entomological Museum of the Universidad del Valle. Colombia may prove to be the most species rich area for *Loneura*.

## Introduction

Recently, García Aldrete et al. (2011b), proposed a classification of *Loneura*, based on the structure of the male hypandrium and phallosome; in it, they recognized three groups of species and assigned the known species in them. A pair of undescribed sister species of *Loneura*, from Valle del Cauca, Colombia, have come since to our attention; they are remarkable in that they constitute a new species group in the genus, and our purpose in this paper is to describe them, and modify the scheme of classification originally presented, to include the new group they represent. The types are deposited in the Entomological Museum of the Universidad del Valle, Santiago de Cali, Colombia (MUSENUV).

## Material and methods

Eight specimens were available for study, three of them were dissected in 80% ethyl alcohol, and their parts (head, right wings, right legs and genitalia), were processed in 100% ethyl alcohol, xilol, clove oil, and mounted on slides in Canada balsam (see González et al. 2011). Before dissection, color was recorded from whole specimens in 80 % ethyl alcohol, observed under a dissecting microscope illuminated with cold white light at 80×. Measurements (given in microns) of parts on the slides were taken with an ocular micrometer, mounted in a Nikon Eclipse 200 microscope. Abbreviation of parts measured are as follows: FW and HW: lengths of right forewing and hindwing, F, T, t1 and t2: lengths of femur, tibia and tarsomeres of right hind leg, ctt1: number of ctenidobothria on t1, Mx4: length of fourth segment of right maxillary palp, f1...fn: lengths of flagellomeres 1...n, of right antenna, IO, D and d, respectively: minimum distance between compound eyes, antero-posterior diameter and transverse diameter of right compound eye, all in dorsal view of head, PO: d/D. The illustrations were made from photographs, taken with a Nikon Coolpix 4500 digital camera, processed in a vector graphics editor CorelDRAW.

## Taxonomy

### (Male) Loneura andina sp. n.

urn:lsid:zoobank.org:act:7039984F-EE31-4FF4-A0B7-A4F9D1968AC1

http://species-id.net/wiki/Loneura_andina

Figures 1–5

#### Type locality.

**COLOMBIA.** Valle del Cauca. Santiago de Cali. Los Andes, Finca Montserrate, 1682 m., 3°25'57.3"N, 76°37'15.4"W.

#### Type material.

Holotype male, 7.IX.2011. On tree trunk covered with mosses, R. González. Deposited in Entomological Museum, Universidad del Valle, Santiago de Cali, Colombia (MUSENUV, slide No. 25548).

#### Etymology.

The specific name refers to the type locality, Los Andes.

#### Diagnosis.

Hypandrium of five sclerites, two side pairs flanking a large central sclerite, this with two large, lateral posterior projections, and two smaller median posterior projections (Fig. 4); phallosome (Fig. 5) Y-shaped anteriorly, external parameres stout, distally rounded, bearing pores; anterior pair of endophallic sclerites stout, bow-shaped, wide anteriorly, pointed posteriorly, with an acuminate projection on outer edge; posterior pair of endophallic sclerites connected anteriorly by a curved bridge, each sclerite long, slender, wide proximally, hooked distally.

**Figures 1–5.**
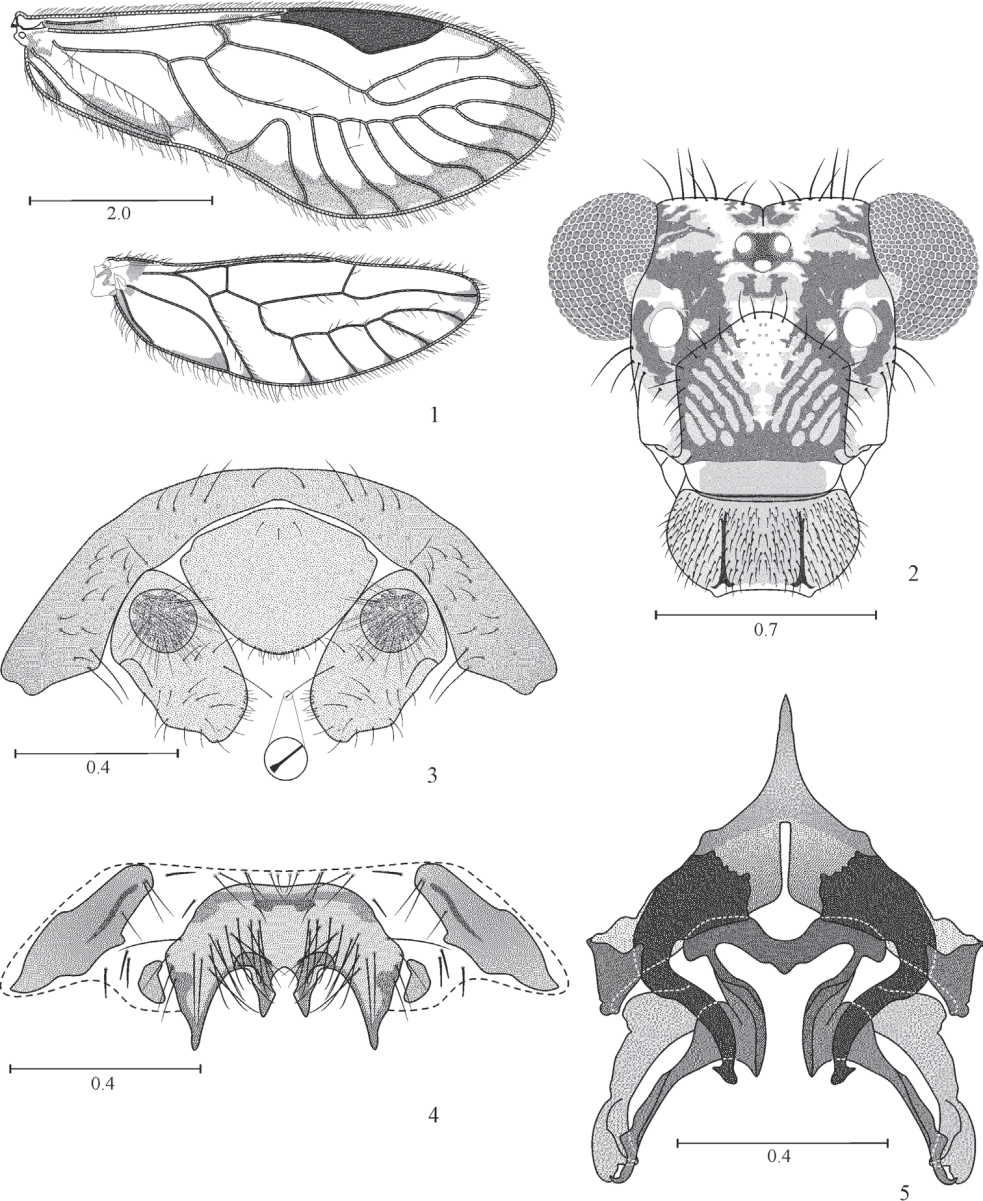
*Loneura andina* sp. n. male **1** Forewing and Hindwing **2** Front view of head **3** Paraprocts and epiproct **4** Hypandrium **5** Phallosome. Scales in mm.

#### Color

(in 80% ethyl alcohol). Body dark brown, with some creamy areas as indicated below. Head with a dark brown oblique band from each compound eye to epistomal sulcus, enclosing the antennal fossae (Fig. 2). Compound eyes black, ocelli hyaline, with ochre centripetal crescents, forming a triangular ocellar group. Vertex creamy white, with a brown spot on each side of the epicranial suture and irregular spots next to each compound eye. Postclypeus with diagonal brown striae. Anteclypeus and labrum brown. Genae creamy white. Antennae: scape brown, pedicel and flagellum pale brown. Mx 1–2 creamy white, Mx 3 brown and Mx 4 brown, with apical third dark brown. Tergal lobes of meso- and metathorax dark brown, thoracic pleura brown, except the metapleura, creamy white. Legs: coxa and trochanter of fore- and mid legs brown; coxa and trochanter of hind leg creamy white, femur of fore- and mid legs with proximal halves brown, distally creamy white; femur of hind leg creamy white, with a subapical brown band; tibiae of all legs brown, distally darker; tarsomere 1 brown, tarsomeres 2–3 dark brown (all legs). Forewings (Fig. 1) hyaline, veins brown, a brown marginal band from R4+5 to distal half of 1A, a brown spot distally on R 2+3, pterostigma dark brown. Hindwings with brown spots distally on the veins (Fig. 1). Abdomen creamy white, with irregular brown spots. Hypandrium yellowish, with sides dark brown; anterior side sclerites dark brown, posterior side sclerites pale brown. Paraprocts and epiproct creamy, with brown spots.

#### Morphology.

As in diagnosis, plus the following: outer cusp of lacinial tip broad, with eight denticles. Forewing pterostigma elongate, widest in the middle; vein M with seven branches; areola postica tall, apically rounded (Fig. 1). Hindwing with M five branched. Paraprocts elongate, setose, each with a macroseta apically dilated, on inner edge, near the apex (Fig. 3); sensory fields with 30 trichobothria on basal rosettes (Fig. 3). Epiproct broadly triangular, wide based, anteriorly convex, posteriorly rounded, with setae as illustrated (Fig. 3).

#### Measurements.

FW: 6025, HW: 4075, F: 1400, T: 2525, t1: 1012, t2: 87, t3: 150, ctt1: 30, f1: 1000, f2: 1075, f3: 950, Mx4: 350, IO: 685, D: 360, d: 495, IO/d: 1.38, PO: 1.3.

### Loneura tuluaensis sp. n.

urn:lsid:zoobank.org:act:5C3BAB7E-9AC7-426F-92BF-34D8445DD1DF

http://species-id.net/wiki/Loneura_tuluaensis

**Figures 6–7.**
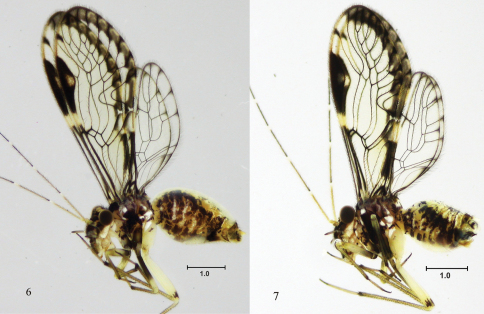
*Loneura tuluaensis* sp. n. Side view **6** female **7** male. Scale in mm.

Figures 6 [Fig F3] 17

#### Type locality.

**COLOMBIA.** Valle del Cauca. Tuluá, Mateguadua, Jardín Botánico Juan María Céspedes, 1127 m, 4°01'29.5"N, 76°09'45.4"W.

#### Type material.

Holotype male. 27.VIII.2011. On tree trunk. Paratypes: 4 females, 2 males, same data as the holotype, on tree and palm trunks. All specimens collected by R. González. Deposited in Entomological Museum, Universidad del Valle, Santiago de Cali, Colombia (MUSENUV, slides 25549-50, vial 25551).

#### Etymology.

The specific name refers to the type locality, Tuluá.

#### Diagnosis.

Male hypandrium of five sclerites, two side pairs, flanking a large central sclerite, this with two large, lateral posterior projections, and two small median posterior projections (Fig. 16). Phallosome (Fig. 17) Y-shaped anteriorly, external parameres elongate, rounded posteriorly, bearing pores; anterior endophallic sclerites bow-shaped, wide anteriorly, slender posteriorly; posterior endophallic sclerites long, slender, distally hooked, wide at base, connected by a broad, triangular bridge. Female ninth sternum (Fig. 12) well sclerotized, with three distinct areas.

#### Female.

**Figures 8–12. F3:**
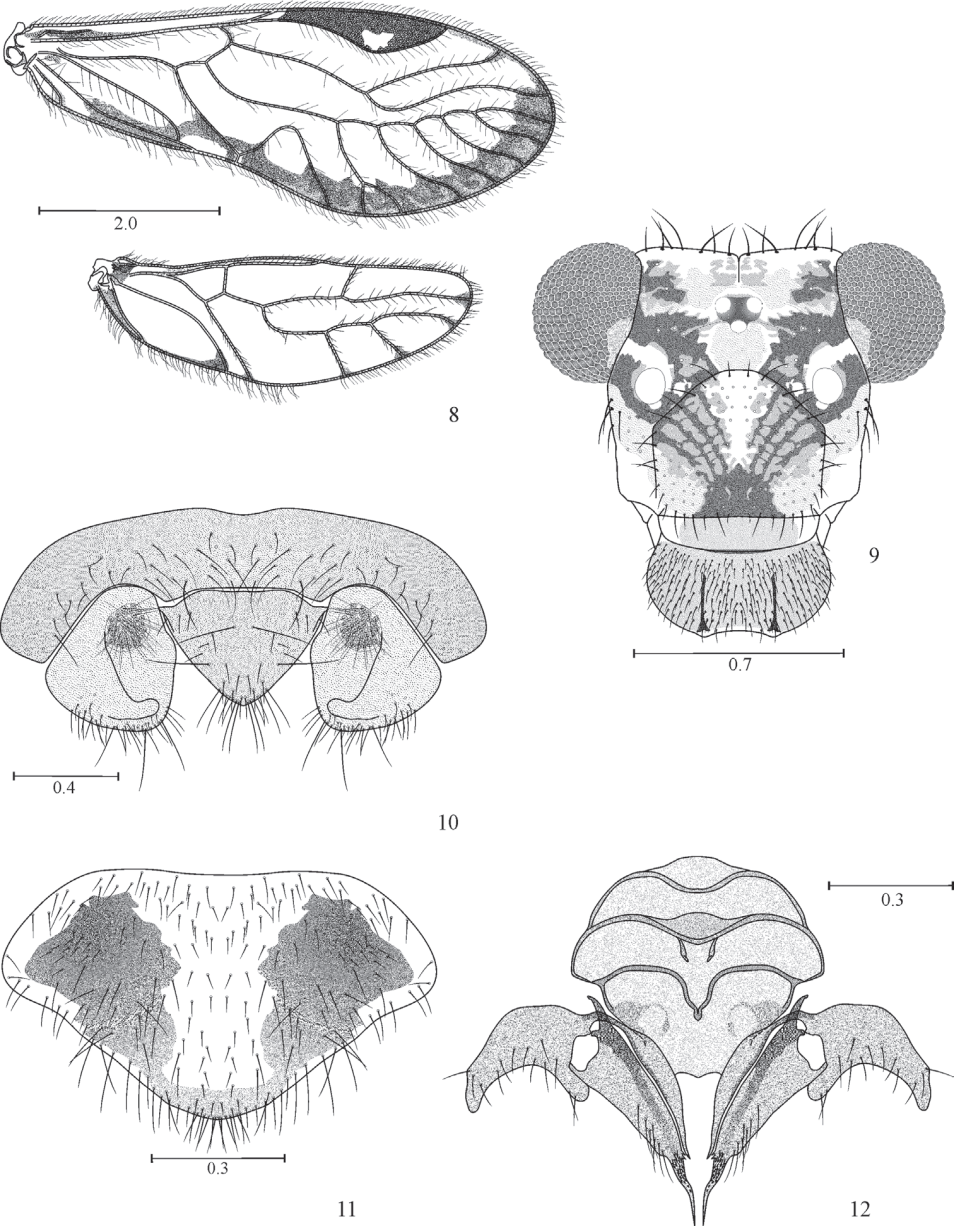
*Loneura tuluaensis* sp. n. female **8** Forewing and Hindwing **9** Front view of head **10** Paraprocts and epiproct **11** Subgenital plate **12** Gonapophyses and ninth sternum. Scales in mm.

**Color** (in 80% ethyl alcohol). Body creamy with brown areas as indicated below (Fig. 6). Head with a wide brown band from each compound eye to epistomal sulcus, enclosing the antennal fossae (Fig. 9). Compound eyes black, ocelli hyaline, with ochre centripetal crescents, forming a triangular ocellar group. Vertex creamy, with brown irregular spots on both sides of epicranial suture and next to each compound eye. Postclypeus with diagonal slender striae. Anteclypeus and labrum pale brown. Genae creamy. Antennae: scape brown, pedicel and first flagellomere pale brown, rest of flagellomeres brown. Mx 1–2 creamy white, Mx 3 brown, Mx 4 brown, with apical third dark brown. Tergal lobes of meso- and metathorax brown, mesothoracic pleura dark brown, pro- and metathoracic pleura creamy white, with brown spots. Legs: coxa and trochanter of fore- and mid legs brown; coxa and trochanter of hind leg creamy white, femur of fore- and mid legs with proximal halves brown, distal halves creamy white; femur of hind leg creamy white, with a brown apical band; tibiae of all legs brown, distally darker; tarsomere 1 of all legs brown, tarsomeres 2–3 of all legs dark brown. Forewings hyaline, with a marginal brown band as illustrated (Fig. 8); pterostigma dark brown, except for a hyaline central area, with brown specks. Hindwings almost hyaline (Fig. 8), vein R 2+3 and branches of M with a brown distal spot. Abdomen creamy (Fig. 6), with brown irregular spots, subgenital plate creamy, middle area hyaline, sides pale brown; gonapophyses brown, IX sternum brown, epiproct and paraprocts creamy white.

**Morphology.** As in diagnosis, plus the following: outer cusp of lacinial tip broad, with 8–9 denticles. Forewings with pterostigma elongate, widest in the middle; vein M with six branches, the last one distally forked; areola postica tall, apically rounded (Fig. 8). Hindwing M with 3–4 branches (Fig. 8). Subgenital plate broad, posteriorly rounded, setae as illustrated (Fig. 11). Gonapophyses (Fig. 12): v1 long, slender, with inner edge more sclerotized, distally acuminate; v2+3 anteriorly heeled, with a group of 6–8 setae on side lobe; distal process long, sinuous, distally acuminate, with a field of minute setae. IX sternum well sclerotized, broadly nut shaped, with three transverse lobes, and well defined borders, as illustrated (Fig. 12). Paraprocts oval, with setae as illustrated (without distally dilated macrosetae); sensory fields with 28 trichobothria on basal rosettes (Fig. 10). Epiproct wide based, broadly triangular, posteriorly rounded, setose as illustrated (Fig. 10).

#### Measurements.

FW: 6025, HW: 4100, F: 1400, T: 2375, t1: 1000, t2: 112, t3: 150, ctt1: 29, f1: 1100, f2: 1125, f3: 975, f4: 962, Mx4: 350, IO: 650, D: 370, d: 480, IO/d: 1.35, PO: 1.29.

#### Male.

**Color.** (in 80% ethyl alcohol). As in the female (Fig. 7), hypandrium yellowish.

**Figures 13–17.**
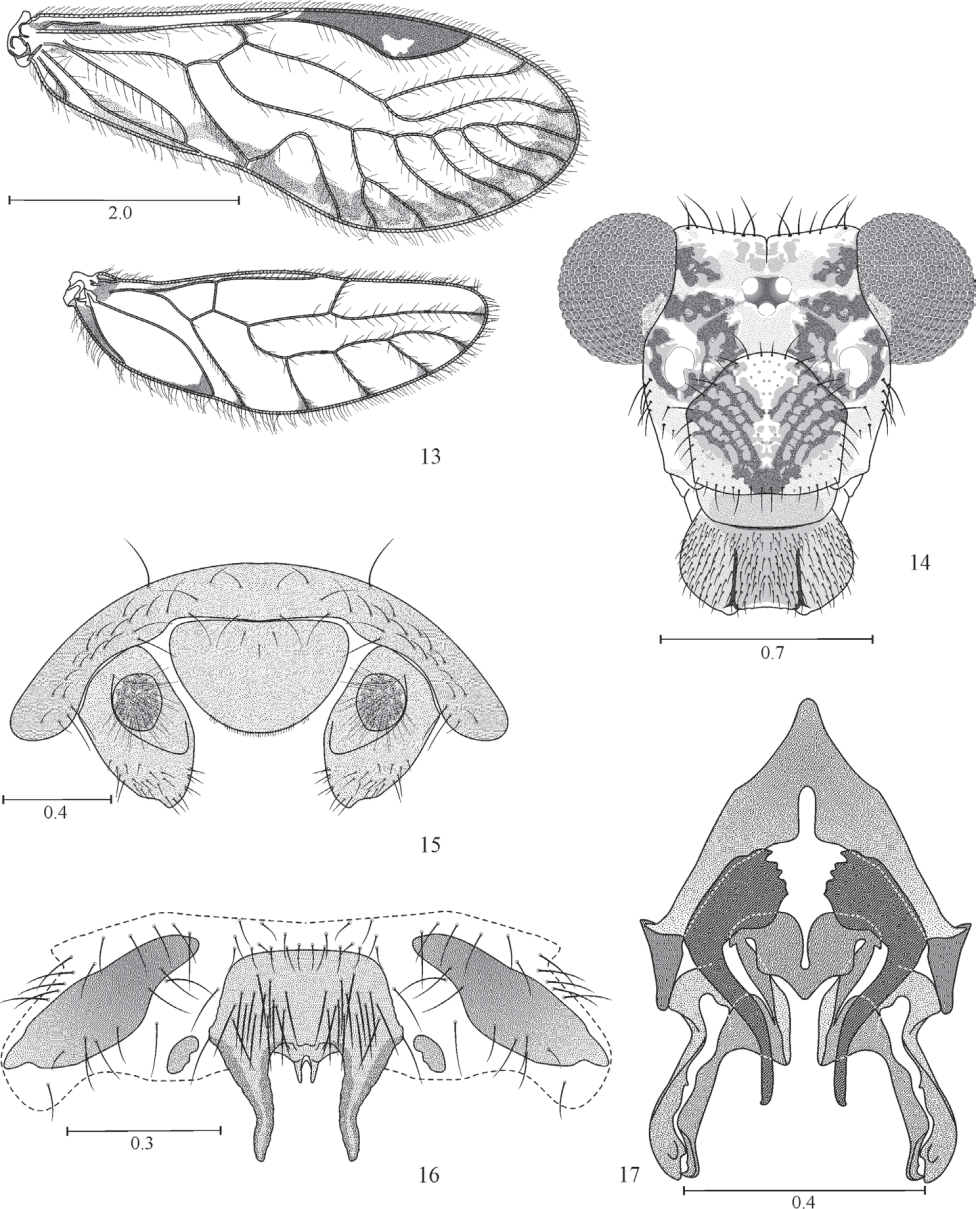
*Loneura tuluaensis* sp. n. male **13** Forewing and Hindwing **14** Front view of head **15** Paraprocts and epiproct **16** Hypandrium. **17** Phallosome. Scales in mm.

**Morphology.** As in diagnosis, plus the following: outer cusp of lacinial tip broad, with nine denticles. Forewings as in the female (Fig. 13). Vein M mostly with six branches, often asymmetrical as in the females (6–6, 6–5, 5–6, or 6–7, for right and left forewings respectively), the last branch forked. Hindwing M four branched, often asymetrical (4–3, 4–4, 5–4, for right and left hindwings respectively). Paraprocts broadly triangular, setae as illustrated (Fig. 15), sensory fields with 30 trichobothria on basal rosettes (Fig. 15). Epiproct broadly triangular, wide based, rounded posteriorly, with setae as illustrated (Fig. 15).

**Measurements.** FW: 5350, HW: 3675, F: 1350, T: 2300, t1: 962, t2: 100, t3: 150, ctt1: 31, f1: 1050, f2: 1100, f3: 950, f4: 800, Mx4: 337, IO: 590, D: 400, d: 530, IO/d: 1.11, PO: 1.3.

#### Discussion.

The two species here described are regarded as sister species based on their similarities in forewing pigmentation pattern, shape of the pterostigma, structure of the hypandrium (constituted of five sclerites, the large central one with two pairs of posterior projections), and on the phallosomes built on the same structural plan (Y-shaped anteriorly, anterior pair of endophallic sclerites bow-shaped, basally wide, and posterior pair of endophallic sclerites slender, distally hooked, anteriorly connected by a broadly triangular bridge).

**Figures 18–21.**
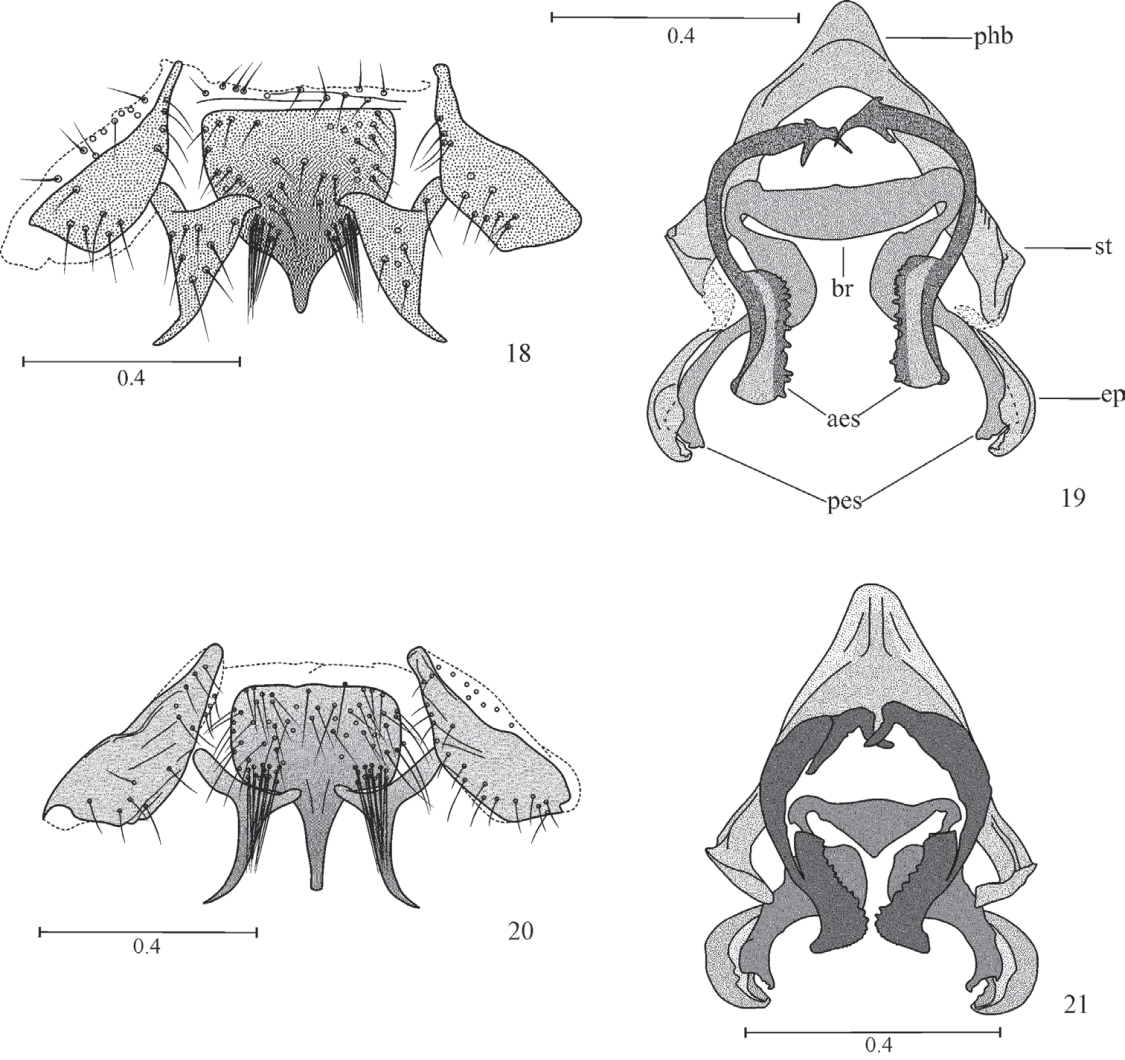
Hypandrium and phallosome **18, 19** *Loneura jinotegaensis* García Aldrete **20, 21** *Loneura mirandaensis* García Aldrete. Scales in mm. (Abbreviations: ep, external parameres; aes, anterior endophallic sclerites; pes, posterior endophallic sclerites; br, bridge; st, side struts; phb, phallobase).

The morphology of the hypandrium and phallosome outlined above impose modifications on Group II, of the infrageneric groups within *Loneura*, proposed by García Aldrete et al. 2011b, as follows:

Group II. Hypandrium consisting of five sclerites, an anterior and a posterior pair, flanking a large central sclerite (Figs 4, 16, 18, 20). Phallosome with external parameres elongate, distally rounded, bearing pores; two pairs of endophallic sclerites, the posterior pair joined proximally by a sclerotized bridge.

Subgroup II a. Anterior side sclerites of hypandrium elongate, posterior side sclerites wide based, elongate, distally acuminate (Figs 18, 20); central sclerite with a median posterior projection, flanked by tufts of macrosetae. Phallosome with external parameres stout, distinctly curved, spoon-shaped; posterior pair of endophallic sclerites curved, acuminate; anterior pair of endophallic sclerites with proximal halves slender, curved, distal halves stout, quadrangular, with a row of blunt teeth along inner edge (Figs 19, 21). Species included: *Loneura jinotegaensis* García Aldrete (Nicaragua), *Loneura mirandaensis* García Aldrete (Venezuela).

Subgroup II b. Anterior side sclerites of hypandrium elongate, posterior side sclerites small, rounded (Figs 4, 16). Central sclerite with four posterior projections, two large on sides, and two small median ones (Figs 4, 16). Phallosome Y-shaped anteriorly, external parameres elongate, distally rounded; anterior pair of endophallic sclerites wide based, bow-shaped, posterior pair of endophallic sclerites slender, distally hooked (Figs 5, 17). Species included: *Loneura andina* García, Mendivil & González (Colombia), *Loneura tuluaensis* García, Mendivil & González (Colombia).

##### Key to males of Loneura Group II species

**Table:** 

1	Central sclerite of hypandrium with one median posterior projection; posterior side sclerites large, wide at base, distally acuminate (Figs 18, 20); posterior endophallic sclerites joined proximally by a stout bridge (Figs 19, 21); Forewing pterostigma with large unpigmented area	[Subgroup II a] 2
–	Central sclerite of hypandrium with four posterior projections, two large lateral ones and two small median ones; posterior side sclerites small, rounded (Figs 4, 16); posterior endophallic sclerites joined proximally by a more slender bridge than above (Figs 5, 17); forewing pterostigma pigmented throughout, at most with small unpigmented area on lower apex	[Subgroup II b] 3
2	Central sclerite of hypandrium with short, pointed median projection (Fig. 18), posterior side sclerites stout, broadly triangular; posterior endophallic sclerites distally slender, bridge straight anteriorly, slightly convex posteriorly (Fig. 19)	*Loneura jinotegaensis* García Aldrete
–	Central sclerite of hypandrium with long, distally truncate median projection, posterior side sclerites wide based, with median projection slender, acuminate (Fig. 20); posterior endophallic sclerites distally stout, bridge triangular (Fig. 21)	*Loneura mirandaensis* García Aldrete
3	Central sclerite of hypandrium with lateral posterior projections wide based, of medium length, acuminate (Fig. 4); median projections long, stout; anterior endophallic sclerites apically with a pointed projection on outer edge (Fig. 5); forewing pterostigma pigmented throughout (Fig. 1)	*Loneura andina* García, Mendivil & González
–	Central sclerite of hypandrium with lateral posterior projections wide based, long, stout, blunt ended; median projections short, slender (Fig. 16); anterior endophallic sclerites apically blunt, without projection as above (Fig. 17); forewing pterostigma with a hyaline area on lower apex (Fig. 13)	*Loneura tuluaensis* García, Mendivil & González

*Loneura* presently includes 46 species, 22 of them undescribed; 21 of the undescribed species are available for study in our collections. Examination of 40 species of *Loneura*, allows us to assert that the following characters are important in distinguishing among the species in the genus:

1. Head pigmentation pattern.

2. Fourth segment of maxillary palps: unpigmented, pigmented throughout or only distally pigmented.

3. Forewings: pattern of pigmentation, number of branches of vein M, branches of M simple or forked.

4. Forewing pterostigma: general shape, pattern of pigmentation or absence of it.

5. Forewing areola postica: general shape.

6. Hindwing: pattern of pigmentation, number of branches of vein M.

7. Legs: pigmentation.

8. Hypandrium: number of sclerites, shape of side sclerites, shape of central sclerite, presence or absence of distinct groups of setae in it, number, position, shape and size of posterior projections.

9. Phallosome: Shape of anterior half, shape and size of external parameres, structure of the anterior and posterior pairs of endophallic sclerites.

10. Female subgenital plate: general shape, setal field, size and shape of side pigmented areas.

11. Ninth sternum: general shape, texture, pigmentation.

12. Gonapophyses: general shape of v1 and v2+3, number and position of setae on v2 lobe, size and shape of v2+3 heel, shape of v2+3 posterior process.

Table 1 presents the geographic distribution of the species known in *Loneura*. The species display a high level of endemism: of the eight Central American species, only two are shared with Mexico and none are shared with South America; of the 28 South American species, one is shared between Bolivia and Argentina, one is shared by Ecuador and Peru, and one is shared by Colombia and Venezuela, the rest are only known in their respective countries, probably a result of insufficient collecting in some areas.

**Table 1.** Geographic distribution of *Loneura* species.

**Area**	**Country**	**No. of species**	**Endemics**
North America	USA	1	1
	Mexico	9	7
	Total	**10**	**8**
Central America	Belize	1	0
	Guatemala	1	0
	Nicaragua	4	2
	Costa Rica	3	2
	Panamá	1	1
	Total	**9**	**5**
South America	Argentina	1	0
	Bolivia	3	2
	Brazil	10	10
	Colombia	11	10
	Ecuador	1	0
	Peru	3	2
	Venezuela	2	1
	Total	**28**	**25**

The species here described raise to 11 the species of *Loneura* known in Colombia, four of them still undescribed (cf. García Aldrete et al. 2011a, b. Ten of the Colombian species are known only from Valle del Cauca (7 species) and from Gorgona Island (3 species), which account for less than 2% of the Colombian territory. Species richness of Colombian *Loneura* is likely much greater than currently documented. Ten additional species are known to occur in Brazil (Moreira de Castro 2007).

## Supplementary Material

XML Treatment for Loneura andina

XML Treatment for Loneura tuluaensis

## References

[B1] García Aldrete AN González Obando R Carrejo NS (2011a) A new *Loneura* from Colombia, and Colombian records of *L. mirandaensis* García Aldrete, and *Loneuroides venezolanus* García Aldrete (Psocodea: ‘Psocoptera’: Ptiloneuridae). Dugesiana 18: 35-37

[B2] García Aldrete AN González Obando R Sarria Sarria F (2011b) Three new species of *Loneura* (Psocodea: ‘Psocoptera’: Ptiloneuridae) from Gorgona Island, Cauca, Colombia, with a new infrageneric classification. Zootaxa 3050: 55-62

[B3] González Obando R García Aldrete AN Carrejo NS (2011) A new species of *Steleops* Enderlein, and a Colombian record of *S. pulcher* New (Psocodea:’Psocoptera’: Psocidae). Zootaxa 2735: 23-27

[B4] Moreira de Castro MC (2007) Revisão taxonômica e filogenia do gênero neotropical *Loneura* Navás, 1927 (Psocoptera: Ptiloneuridae). Dissertacao (mestrado) - Area de concentracao Entomologia – INPA/UFAM, Manaus, Amazonas, Brasil, 67 p. + 61 figuras.

